# First-Time Successful Trial Without Catheter (TWOC) After GreenLight Laser Photoselective Vaporization of the Prostate (GLL PVP) Surgery for an Enlarged Prostate

**DOI:** 10.7759/cureus.72982

**Published:** 2024-11-04

**Authors:** A B Azharul Islam, Maisha Zaman Poushi

**Affiliations:** 1 Urology, West Middlesex University Hospital, London, GBR; 2 Surgery, Dhaka Medical College and Hospital, Dhaka, BGD

**Keywords:** enlarged prostate, greenlight laser pvp, long-term catheter, successful twoc, trial without catheter, twoc

## Abstract

Objective

We aimed to assess the success rate of the GreenLight laser photoselective vaporization of the prostate (GLL PVP) procedure for enlarged prostates in patients with and without preoperative long-term catheters.

Methodology

A retrospective data analysis was conducted on 46 patients. Data were collected from clinical health records and radiology images. The parameters studied in this analysis included the patient's age, prostate volume, medical management before surgery, and whether the patient had a catheter or not prior to the surgery.

Results

A total of 46 GLL PVP surgeries were performed over a 12-month period from May 2023 to May 2024 at West Middlesex University Hospital in London. Patients had a mean age of 73.2 ± 8.12 years, and their prostate volume was 57.7 ± 25.96 cc. Before the surgery, 14 cases (30%) were using long-term catheters, and 32 cases (70%) were not. Additionally, 40 cases (87%) were receiving medical management for lower urinary tract symptoms (LUTS). After the surgery, 39 cases (85%) passed their first trial without catheter (TWOC), and seven cases (15%) failed it, regardless of whether they had used catheters before the surgery. Patients using long-term catheters before the operation had a 71% success rate in passing their first TWOC and a 29% failure rate.

Conclusion

Our study found that GLL PVP has a significant positive impact on elderly individuals with a large prostate volume, regardless of whether they had long-term catheters or not prior to the operation. Additionally, our findings indicate that patients without preoperative long-term catheters experience significantly better outcomes.

## Introduction

Benign prostatic hyperplasia (BPH) is a non-malignant enlargement of the prostate gland due to cellular proliferation. This condition can result in urinary manifestations such as frequent or painful urination, incontinence, a diminished urine stream with hesitancy, difficulty initiating urination, and an elevated susceptibility to urinary tract infections. BPH affects 50-60% of men aged 60-70 years, with this prevalence rising to 80-90% among men aged 70-80 years [1].

The initial treatment involves using alpha-blockers, 5-alpha-reductase inhibitors, and anticholinergics either alone or in combination. Clinical trial results indicate that combination therapy significantly enhances symptom scores and peak urinary flow compared to either monotherapy option. Additionally, combining medications lowers the risk of urinary retention and the need for prostate surgery. However, some patients may not find sufficient relief from these medications and may require surgical intervention to alleviate bladder outlet obstructions [2,3].

The minimally invasive surgical treatments for lower urinary tract symptoms (LUTS) caused by BPH are transurethral resection in saline (TURIS) or vaporization techniques using plasma kinetic or laser energies. These procedures have been found to be more convenient than transurethral resection of the prostate (TURP) in terms of postoperative complications such as bleeding, urethral strictures, urinary incontinence, and transurethral resection (TUR) syndrome [4].

Laser therapies provide a new approach to treating BPH, and GreenLight laser photoselective vaporization of the prostate (GLL PVP) is increasingly being researched as a potential primary treatment [5-8]. This technique typically involves using a 532 nm green laser generated with potassium-titanyl-phosphate (KTP) or lithium triborate crystals [9]. Unlike other types of lasers, the green laser is easily absorbed by soft tissue hemoglobin, leading to improved coagulation and reduced risk of deeper tissue injuries during vaporization [10,11].

GLL PVP has been proven to be a safe and effective treatment option [12]. According to the GOLIATH trial, GLL PVP is as effective as TURP in treating this condition [13].

We aimed to assess the success rate of the GLL PVP procedure for enlarged prostates in patients with and without preoperative long-term catheters.

## Materials and methods

This study took place at the Urology Department of the West Middlesex University Hospital in London, United Kingdom, over a period of 12 months from May 2023 to May 2024. The study included all consecutive patients who underwent GLL PVP to treat LUTS associated with BPH.

The parameters examined in this study included the patient's age, prostate volume, medical management before surgery (alpha-blockers and 5-alpha-reductase inhibitors), and whether the patient had a long-term catheter or history of chronic urinary retention prior to the surgery.

Retrospective cohort studies were conducted, and data were collected from patients' clinical health records and radiology images.

## Results

From May 2023 to May 2024, 46 men with BPH-induced LUTS underwent the GLL PVP. The demographic and preoperative characteristics of the patients are presented in Table 1.

**Table 1:** Patient demographics and preoperative characteristics

Parameters	Mean ± Standard Deviation	
Age (years)	73.2 ± 8.12	
Prostate volume (cc)	57.7 ± 25.96	
Medical management before the operation	Yes, 40 (87%)	No, 6 (13%)
Long-term catheter or h/o urinary retention before the operation	Yes, 14 (30%)	No, 32 (70%)

**Figure 1:**
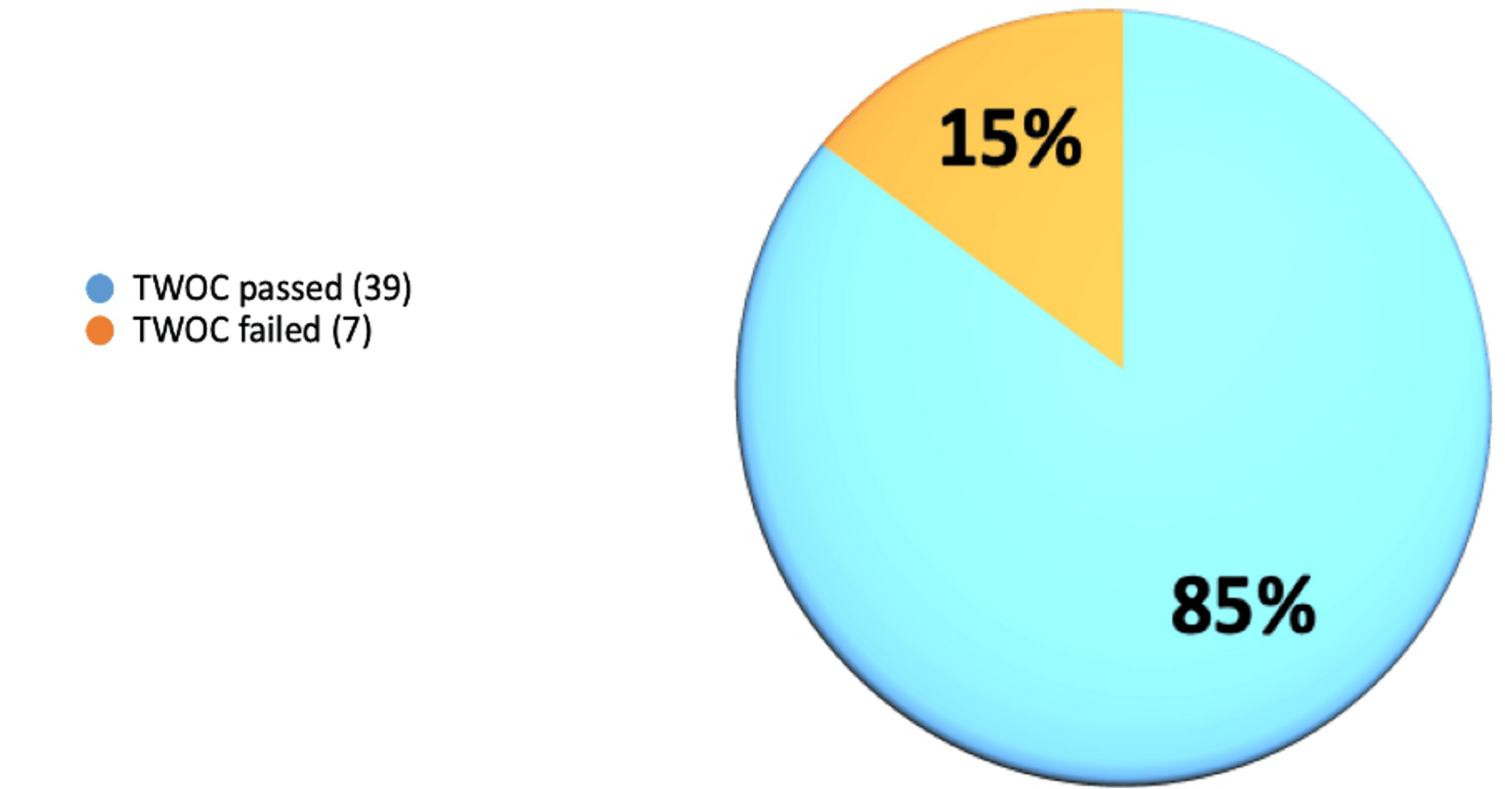
First-time successful TWOC for patients with and without preoperative long-term catheters TWOC: trial without catheter

**Figure 2:**
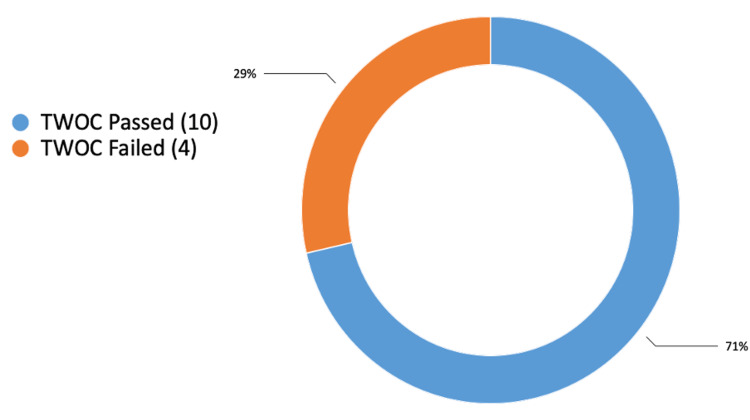
First-time successful TWOC for patients with long-term catheters due to chronic urinary retention TWOC: trial without catheter

**Figure 3:**
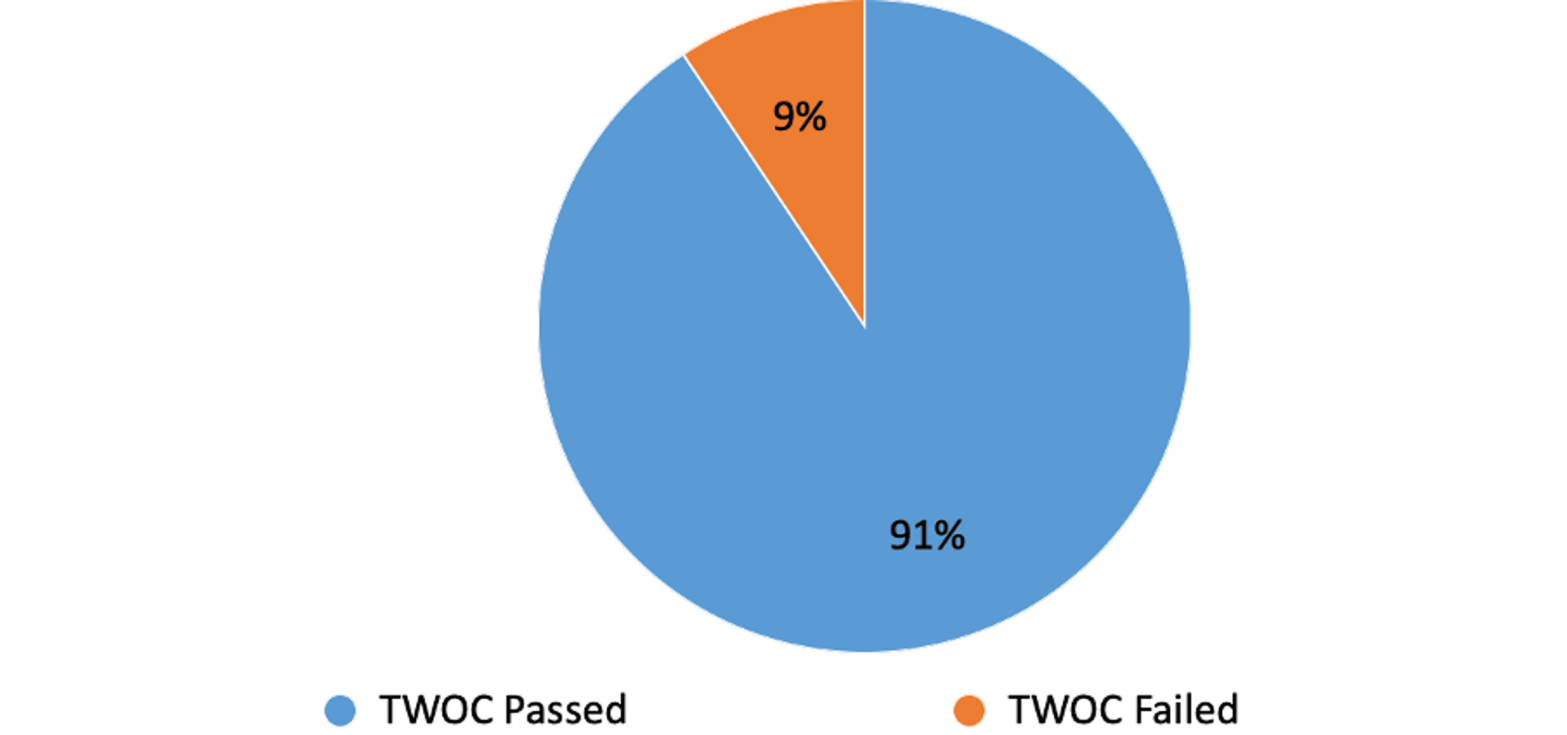
First-time successful TWOC for patients without long-term catheters preoperatively TWOC: trial without catheter

On the first postoperative day after surgery, 39 cases (85%) passed their first trial without catheter (TWOC), and seven cases (15%) failed it, regardless of whether they had used catheters before the surgery (Figure 1). Patients using long-term catheters for chronic urinary retention before the operation had a 71% success rate in passing their first TWOC and a 29% failure rate (Figure 2). Patients without long-term catheters before the operation had a 91% (29 cases) success rate in passing their first TWOC, and the failure rate was 9% (three cases) (Figure 3). For the patients who did not succeed TWOC, the average post-void residual (PVR) volume was 450 mL.

## Discussion

The results of this study indicate that 85% of patients successfully passed their first TWOC on the first postoperative day, while 15% did not, regardless of prior catheter use or history of urinary retention. An important deduction from the study is that patients with chronic urinary retention and long-term catheters have shown notable improvement in passing urine following GLL PVP operation. Patients who used long-term catheters before the operation achieved a 71% success rate in passing their initial TWOC and a 29% failure rate. Additionally, it is noteworthy that a significant outcome was noticed in the patients with no urinary retention or long-term catheter prior to the operation, with a 91% success rate in passing their first TWOC.

GreenLight laser has unequivocally demonstrated its effectiveness in alleviating the LUTS linked to BPH. Compelling evidence indicates that, in comparison to TURP, GLL PVP is linked to significantly shorter hospital stays, reduced postoperative catheterization, and notably higher preservation of ejaculatory function at 12 months. Leading clinical experts have emphatically confirmed that, based on their extensive experience, GreenLight laser operation stands as a highly effective treatment option for individuals with BPH [14].

In the context of high-risk groups such as those with urinary retention, prostates exceeding 100 mL in volume, and a heightened risk of bleeding, there is substantial clinical evidence to support the assertion that GLL PVP is comparably effective to TURP in the management of BPH symptoms [14].

The GreenLight laser procedure is considered safe for treating prostates with a volume of up to 100 mL. Additionally, it has been determined that prostates up to 150 mL can be appropriately treated with GLL PVP under the care of an experienced clinician [14].

Research indicates that up to 75% of patients in need of treatment for an enlarged prostate could be managed on an ambulatory care basis. Studies comparing GreenLight with TURP have proven the effectiveness of photoselective vaporization of the prostate [15,16]. The GOLIATH study, a randomized, multicenter, non-inferiority trial, has compared the GLL PVP with TURP. Results have shown comparable effectiveness in terms of the International Prostate Symptom Score (IPSS), maximum urinary flow rate, and residual urine at six and 12 months [15,16].

Limitations of the study

Our study provides valuable insights into the success rate of the GLL PVP procedure for BPH regarding patients passing their first TWOC. However, it is important to acknowledge some limitations. The primary limitation is the retrospective nature of our analysis. In addition, we need to include more demographic features, such as whether the patient had any preoperative high-risk factors, including ASA (American Society of Anesthesiologists) grading. The small sample size of our study may restrict the generalizability of the results and may not adequately represent the broader population. It is also crucial to follow up with patients to determine if they developed urinary retention after passing their first TWOC. To gain a more comprehensive understanding, a larger multicentric prospective study would be much more representative, especially among the diverse demographic of BPH patients. Future research should focus on conducting prospective studies with larger sample sizes and collaborative efforts across multiple centers.

## Conclusions

Our comprehensive study demonstrates that GLL PVP has a significant positive impact on elderly individuals with a large prostate volume, irrespective of whether the patients had long-term catheters or not before the operation. Furthermore, our findings assert that patients without preoperative long-term catheters or a history of chronic urinary retention exhibit significantly better outcomes. Therefore, it is evident that GLL PVP is a safe and highly effective procedure for an enlarged prostate, leading to rapid improvement in both subjective and objective voiding parameters.
